# 

**Published:** 1838-01-17

**Authors:** 


### TERMS.

The Examiner is published every alternate Wednesday, on a super-royal sheet at *Three dollars* per annum, *in advance.* Each number contains sixteen octavo pages of double column, making an annual volume of upwards of four hundred pages, furnished with an index and title page.

Letters and communications may be addressed to the "Editors of the Medical Examiner," and must be *post paid, otherwise they will not be taken from the office.*

Orders for subscription, accompanied with a remittance, will be met by prompt attention.

Persons enclosing *Five* dollars, will be entitled to *two copies, if sent to the same address.*

Advertisements are inserted at the rate of $1 for 12 lines.

Office No. 230 Spruce street, between Sixth and Seventh streets.

This No. contains 1½ sheets.

